# Reconstruction of auricular conchal defects with local flaps

**DOI:** 10.1097/MD.0000000000005282

**Published:** 2016-11-18

**Authors:** Ji Zhu, Hui Zhao, Kai Wu, Chuan Lv, Hong-da Bi, Meng-yan Sun, Yu-chong Wang, Xin Xing, Chun-yu Xue

**Affiliations:** Department of Plastic Surgery, Changhai Hospital, Second Military Medical University, Shanghai, China.

**Keywords:** auricular conchal cavity, island flap, skin defect

## Abstract

Reconstruction of the auricular conchal cavity is relatively difficult because of its unique structure, shape, and location. We compared different methods of repair of the auricular concha to determine the method that would cause the least injury to the donor site.

The method selected was based on the location and size of the defect. If the defect was located in the upper part of the concha, or if the defect was >15 mm in diameter, we used a post-auricular subcutaneous pedicle island flap that was pulled through a post-auricular sulcus tunnel to cover the wound. If the defect was located in the lower part of the concha and was <15 mm in diameter, we used a pre-auricular translocation flap that was passed through the intertragic notch to cover the wound. The donor site was closed primarily. All flaps survived well and any scars associated with the surgery were unnoticeable. No tumor relapse or metastasis was observed over a mean follow-up period of 35 months. All patients were satisfied with the outcome.

The periauricular flap technique chosen for reconstruction of skin defects in the auricular concha depends on the size and location of the defect. With appropriate flap selection, excellent functional, and aesthetic outcomes are achieved.

## 1 Introduction

The auricular concha refers to the central portion of the external ear. It is composed of thin skin, subcutaneous tissue, and concave shaped cartilage. Reconstruction of the concha is performed for esthetic reasons as well as to maintain the structural firmness of the cavum conchae, an important structure contributing to the conduction of sound waves to the external auditory canal.

Flaps,^[1–6]^ partial-thickness skin grafts, and full-thickness skin grafts have been used for reconstruction of auricular defects. The retroauricular region is the principal donor site for reconstruction of defects of the ear.^[7–9]^ Most of the methods described have been for reconstruction of the upper, middle, or lower third of the ear. Defects of the central portion of the ear are less common and only a few surgical techniques have been described.^[10]^

In general, skin grafts are prone to delayed wound healing, pigmentation, and centripetal contraction, which can lead to deformation of the conchal cavity, affecting functional and esthetic outcomes.^[3]^

This article describes the technique the authors used to reconstruct skin defects of the cavum conchae using either modified postauricular subcutaneous pedicle island flaps or pre-auricular local flaps, achieving excellent reconstructive outcomes.

## 2 Methods

### 2.1 Preoperative evaluation and treatment

Twenty-one patients presenting with tumors located on the anterior conchal surface, who were treated at the department of Plastic Surgery of Changhai Hospital (Shanghai, China) from 2010 to 2014, were included in this analysis. The patient distribution included 10 men and 11 women, with ages ranging from 25 to 64 years. The mean age was 53.5 years. The tumor distribution of the 21 patients included: 13 patients with basal cell carcinomas, 3 patients with squamous cell carcinomas, 3 patients with seborrheic keratosis, and 2 patients with pigmented nevus.

In all of the patients, the lesions were excised under local anesthesia. Appropriate margins were obtained based on the tumor behavior and the size of the lesion. All resected specimens were submitted for pathological analysis in order to determine a histologic diagnosis. Flap reconstruction was performed at the time of tumor excision in all of these cases. Informed consents were given before surgery. The surgical procedure and this report have been approved by the ethics committee of Changhai Hospital of Shanghai.

### 2.2 Surgical technique and postoperative management

**Figure 1:**
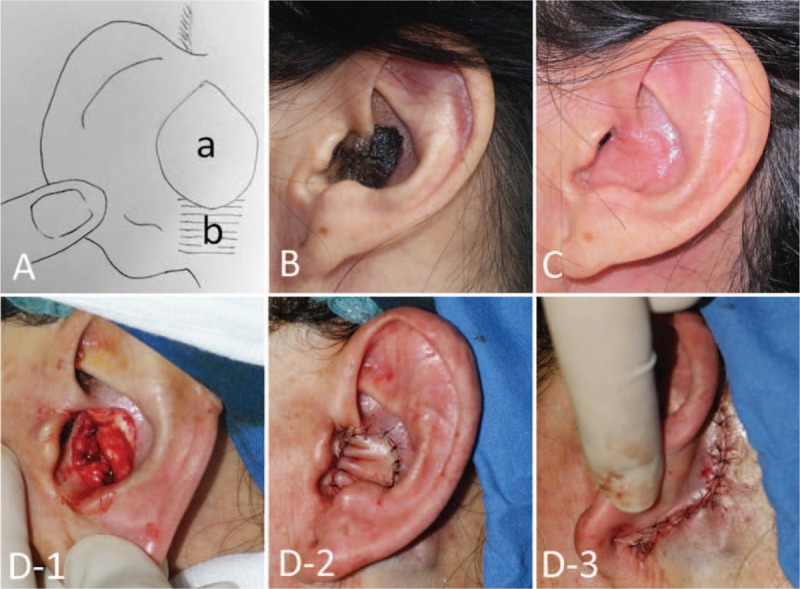
(A) Design of postauricular island pedicle flap. (a) Island flap; (b) subcutaneous pedicle. (B) Before surgery; (C) 2 months after surgery; (D) Intraoperative: (D-1) removal of tumor; (D-2) frontal view immediately after surgery; (D-3) postauricular view immediately after surgery.

The postauricular subcutaneous pedicle island flap **(**PASPI) was used for repair of defects located in the upper part of the concha, or for defects that were >15 mm in diameter. The vertical axis of the flap was created along the sulcus auriculae posterior. The subcutaneous pedicle was created in the lower third of the ear. The tissue of the postauricular subcutaneous pedicle island flaps was created to be slightly larger than the area of the defect, ranging from 1.5 cm × 1.5 cm to 2.5 cm × 3.0 cm. The length of the pedicle was approximately 1 cm to 1.5 cm. A spindle-shaped skin incision was made, taking care to preserve a portion of the skin over the pedicle (Fig. 1). The flap was tongue shaped, with a length to width ratio ranging from 2:1 to 5:1. A slit was created along the postauricular sulcus by resecting part of the cartilage. The flap was pulled through the slit onto the anterior surface of the concha. The slit was created to be wide enough to allow the flap to pass without resistance, reducing the risk of ischemia. The postauricular donor site was closed through primary skin closure, using an interrupted suture pattern (Fig. 1). The sutures were removed after 1 week.

**Figure 2:**
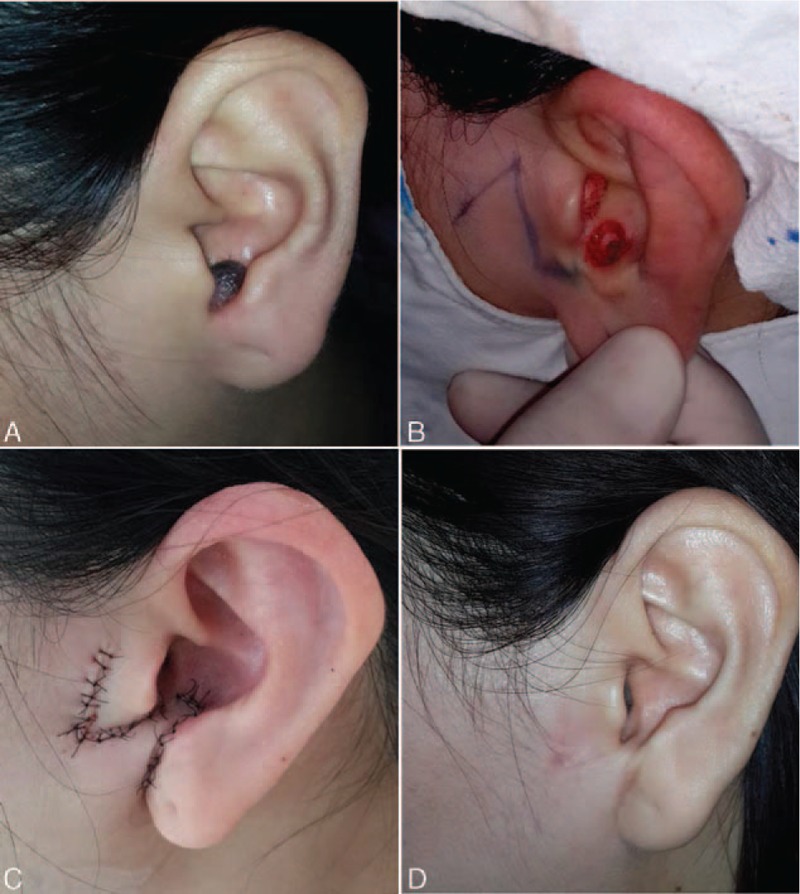
(A) Before surgery; (B) Intraoperative: design of preauricular translocational flap; (C) immediately after surgery; (D) 6 months after surgery.

Defects that were located in the lower part of the concha and were < 15 mm in diameter, were repaired using a preauricular translocation flap (PAT). A translocation flap was created in front of the tragus, with the pedicle situated inferiorly and the distal end of the flap situated superiorly. The skin of the intertragic notch, between the defect and the flap, was excised. The flap was rotated through the intertragic notch in order to cover the defect (Fig. 2). The skin of the donor site was closed with a primary closure technique through mobilization of the surrounding tissue. The sutures were removed after 1 week.

## 3 Results

Thirteen defects were reconstructed using PASPIs and seven defects were reconstructed using PATs. Histopathology reports confirmed the diagnosis of malignant tumors, with complete excision of the lesions in all cases. Follow-ups ranged from 15 months to 51 months, with a mean of 35 months. No complications occurred. Potential complications included: flap necrosis, infection, recurrence, or metastasis. Donor-site scars were unnoticeable; the color and texture of the flaps matched the surrounding skin well. Aesthetic outcomes were good, no auricular deformation occurred, and hearing was not affected in any case.

## 4 Discussion

Reconstruction of defects of the auricular concha is difficult because the skin in this region is closely adhered to the underlying cartilage. Many surgeons still use skin grafting for repair of defects in this region. However, skin grafts may be associated with complications such as delayed wound healing, pigmentation, and centripetal contraction.^[3]^ Pigmentation is especially obvious in oriental patients. In order to decrease the risk of these complications, the flaps for conchal reconstruction should be considered as an alternative repair technique.

When performing reconstruction of the ear, care should be taken to avoid meatal stenosis, distortion of the concha, or significant esthetic change as much as possible. The posterior auricular skin is relatively flexible, and the auriculotemporal angle is 30° to 60°, which permits a skin flap to be created on the anterior auricular surface if the auricle is pulled close to the skull.

The main vascular supply to the auricle is via the superficial temporal artery and the posterior auricular artery. The posterior auricular artery ascends behind the ear in the auriculocephalic sulcus and branches into 3 to 5 sizable vessels providing blood supply to the lower, middle, and upper posterior auricle.^[11–13]^

Many surgeons have used postauricular flaps for reconstruction of the auricular region. The advantages of the postauricular flap include: minimal donor site morbidity; cohesion of the flap skin color, thickness, and texture; and inconspicuous scar formation.^[9,10,14,15]^ The donor site can be closed by primary intention through mobilization of the surrounding tissue. Masson described the conventional postauricular “revolving-door” island flap for ear reconstruction in 1972.^[16]^ Refinements to the original technique have since been described.^[4,5,17]^ Ghassemi described the anterior pedicle retroauricular flap for reconstruction of full-thickness defects in different parts of the ear,^[10,14]^ and McInerney described the “trap-door flap” as a reliable and reproducible method for ear reconstruction.^[18]^ Despite the variety of repair techniques available, only a few methods have been developed for defects in the central region of the auricle.

After a thorough review of the anatomy of the auricle, we developed a simplified procedure for the PASPI for conchal reconstruction. In addition to the advantages mentioned earlier, there is no need to search for an arterial supply in this location, which simplifies the procedure and decreases the risk of trauma to the donor site. Furthermore, part of the skin over the pedicle is preserved during surgery. This reduces suture line tension and the risk of secondary deformity, eliminating the requirement for skin grafting. In order to avoid a “trap-door like deformity,” a thin subcutaneous layer with a gliding interface is created at the periphery of the flap.

The PAT is located in the preauricular region of the face and is supplied by the branches of the superficial temporal artery. It is especially suitable for repair of defects of the lower cavum conchae. The advantages of the preauricular translocation flap are as follows: there is less trauma; the procedure is simple; the flap has a rich blood supply; the flap survival rate is high; and the natural shape of the auricle is preserved. The donor site can be closed primarily. Compared to the PASPI reconstruction, the range of the preauricular flap is limited and the scar at the donor site is relatively obvious. Moreover, since the pedicle is close to the intertragic notch, the preauricular flap is more appropriate for repair of defects in the lower part of the conchal cavity; for defects in the upper part of the concha, this flap is not long enough and is not the best choice.

When small defects are present, some patients select to allow their wounds to heal through secondary epithelization.^[1]^ Secondary epithelialization can be associated with severe hypertrophic scarring, which can lead to poor function and aesthetic outcomes. Therefore, in our opinion, immediate repair is advisable for auricular defects.

## 5 Conclusion

We consider the retroauricular island flap and the preauricular translocation flap to be excellent choices for repair of auricular conchal defects. The 2 methods can be used either alone or in combination, depending on the defect size and location, to achieve excellent reconstructive outcomes.
